# In-context adaptation of VLMs for few-shot cell detection in optical microscopy

**DOI:** 10.3389/frai.2026.1761903

**Published:** 2026-05-15

**Authors:** Shreyan Ganguly, Angona Biswas, Jaydeep Rade, Md Hasibul Hasan Hasib, Nabila Masud, Nitish Singla, Abhipsa Dash, Ushashi Bhattacharjee, Soumik Sarkar, Aditya Balu, Anwesha Sarkar, Adarsh Krishnamurthy

**Affiliations:** Iowa State University, Ames, IA, United States

**Keywords:** artificial intelligence, biomedical imaging, few-shot learning, microscopy, multimodal sensing, object detection, reasoning

## Abstract

Foundation vision-language models (VLMs) excel on natural images, but their utility for biomedical microscopy remains underexplored. In this paper, we investigate how in-context learning enables state-of-the-art VLMs to perform few-shot object detection when large annotated datasets are unavailable, as is often the case with microscopic images. We introduce the Micro-OD benchmark, a curated collection of 252 images specifically curated for in-context learning, with bounding-box annotations spanning 11 cell types across four sources, including two in-lab expert-annotated sets. We systematically evaluate eight VLMs under few-shot conditions and compare variants with and without implicit test-time reasoning tokens. We further implement a hybrid Few-Shot Object Detection (FSOD) pipeline that combines a detection head with a VLM-based few-shot classifier, which enhances the few-shot performance of recent VLMs on our benchmark. Across datasets, we observe that zero-shot performance is weak due to the domain gap; however, few-shot support consistently improves detection, with marginal gains achieved after six shots. We observe that some reasoning variant models show task-specific gains, but the effect varies across models and settings. Our results highlight in-context adaptation as a promising research direction requiring further development for microscopy, and our benchmark provides a reproducible testbed for advancing open-vocabulary detection in biomedical imaging. Our project page is at: here.

## 1 Introduction

Deep Learning (DL) has revolutionized the field of biomedical imaging by enhancing or automating several imaging-based workflows. This enhancement has spanned diverse modalities, including radiology (CT, MRI, ultrasound) and microscopy (optical microscopy, photoacoustic imaging, and label-free, live-cell imaging) (Zhou et al., 2021). Among these, microscopy is a cornerstone of modern biology and medicine, providing critical insights into cellular structures and mechanisms essential for disease diagnosis, treatment monitoring, and basic biological discovery (Weissleder and Nahrendorf, 2015). However, manual interpretation of microscopy images is time-consuming, subjective, and impractical for large-scale studies such as high-throughput screening or clinical trials (Gurcan et al., 2009). DL addresses these limitations by automatically extracting hierarchical and fine-grained features from complex, high-dimensional data (Zhou et al., 2021). This capability enables scalable interpretation of microscopy images for clinical and biological applications. A key example is the detection and localization of individual cells, which supports quantification of morphology, density, and dynamics. These measurements are central to fields such as developmental biology, cancer, immunology, and neuroscience (Caicedo et al., 2017). High-content microscopy screens further rely on robust cell detection to evaluate treatment effects, toxicity, and drug efficacy at the single-cell level (Smith et al., 2022). These demands for precision, scalability, and objectivity have driven the adoption of DL-based AI pipelines in microscopy image analysis.

While DL has transformed microscopy image analysis, earlier attempts relied heavily on classical computer vision techniques, such as thresholding, morphology, and template matching, for analyzing cells, lesions, or anatomical structures (Suzuki, 2017). While effective in restricted settings, these approaches rely on hand-tuned heuristics and degrade when there are shifts in staining, illumination, or instrumentation. Modern, fully supervised deep detectors, such as Faster R-CNN (Ren et al., 2015) or YOLO (Redmon and Farhadi, 2018), deliver high accuracy when large, high-quality labels are available. Still, they remain constrained by their training dataset, require costly fine-tuning to cover new categories, and are brittle under domain shifts common in microscopy (Zou et al., 2023; Litjens et al., 2017; Shen et al., 2017). Task-specific training pipelines are expensive (in terms of annotation and computation), slow to adapt (to new cell types/protocols), and semantically closed-world (they cannot recognize unseen classes without retraining).

Few-Shot Object Detection (FSOD) (Han and Lim, 2024) has offered a pathway to adapt powerful pre-trained models to novel domains with less supervision. While FSOD methods mitigate supervision scarcity, they still depend on complex meta-learning or fine-tuning pipelines and remain limited across various domains. Open-Vocabulary Object Detection (OVD) (Minderer et al., 2022) extends this idea by aligning visual features with language, enabling detectors to recognize unseen categories from text prompts without any specific training (Zhang et al., 2024). Building on OVD, Foundation models like Grounding DINO (Liu et al., 2024) and OWL-ViT (Minderer et al., 2022) exemplify this trend, leveraging large-scale pretraining to localize and classify a wide range of objects described by text prompts.

The advent of foundation models has revolutionized the paradigm of computer vision, particularly Vision-Language Models (VLMs) that are pre-trained on web-scale multimodal data. By utilizing the extensive prior knowledge embedded in foundation models, it is possible to quickly adapt to new object categories with only a limited number of annotated examples. This process is referred to as in-context learning (ICL) (Zhou et al., 2022). Generalist models such as GPT (Achiam et al., 2023), Gemini (Comanici et al., 2025), and Claude (Kurokawa et al., 2024) are trained on enormous datasets of image-text pairs to learn a joint embedding space, where semantically related visual and textual features are mapped close to one another. This alignment enables VLMs to be prompted with natural language descriptions, allowing them to recognize and localize novel categories, even if those categories were not present in the original training data. The use case of these generalized models can be extended to detecting arbitrary objects in photos, leveraging natural language prompting. However, it is observed that the detection performance of these generalist models is confined to images that tend to be more frequent on the internet, and does not generalize to the tail of the distribution of the pretraining data (Robicheaux et al., 2025).

**Figure 1:**
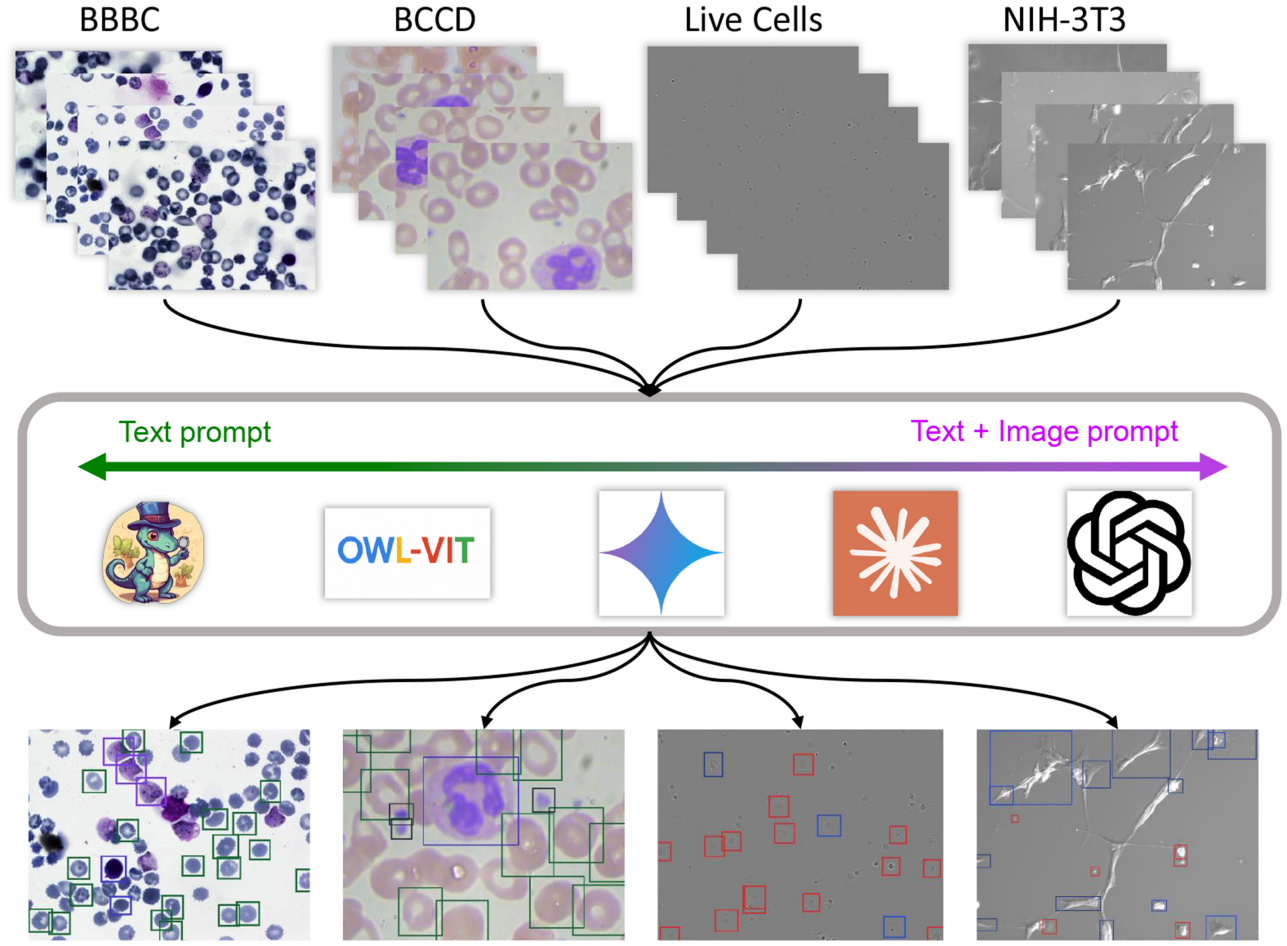
An overview of the Few-Shot Object Detection (FSOD) pipeline using vision-language models (VLM) for cell shape detection. We process four different microscopy datasets (BBBC, BCCD, Live Cells, and NIH-3T3) by various VLMs using either a text-only or a combined text and image prompt to generate bounding box detections for different cell types.

Biomedical microscopy is a relatively underrepresented domain in internet-scale datasets and presents distinctive vision challenges, such as fine-grained visual differences between classes, low contrast, and frequent imaging artifacts (Lozano et al., 2024). These factors create a substantial domain shift for models pre-trained on web-scale natural images. The diversity of imaging techniques further widens this gap; for instance, the stained appearance of bright-field microscopy is significantly different from the grayscale, halo-laden images produced by phase-contrast microscopy. These challenges make VLMs especially attractive for biomedical microscopy, where new cell types or imaging conditions often fall outside any pretraining distribution. Yet, despite their promise of prompt-driven generalization, their effectiveness in this domain remains underexplored. To address this gap, we introduce Micro-OD, a curated benchmark for microscopy-specific evaluation under in-context learning, and conduct a systematic study. An overview of the FSOD pipeline and evaluation workflow is shown in Figure 1.

Our main contributions include:

- Micro-OD, a curated benchmark for few-shot object detection in microscopy. It comprises 252 images with bounding-box annotations for 11 cell types, sourced from public and in-house datasets (BCCD Shenggan, 2017, BBBC Ljosa et al., 2012, LIVECell Edlund et al., 2021, and NIH-3T3).
- Comprehensive evaluation of eight state-of-the-art VLMs under zero-shot and few-shot (K=1,3,6) conditions. Our analysis uniquely compares variants with and without implicit reasoning ("thinking") tokens to assess their impact on localization and classification tasks.
- Validation of a hybrid FSOD pipeline that decouples localization from classification. This cascaded approach proves highly effective, significantly outperforming end-to-end methods and reaching a top mF1 score of 0.30, a fivefold increase over the best zero-shot baseline.

Together, these contributions provide the first comprehensive study of VLMs for few-shot cell detection in microscopy, offering insights into the limitations and opportunities of adapting foundation models to biomedical domains.

## 2 Related work

### 2.1 Vision language foundation models for object detection

Fully supervised object detectors perform well in closed-set scenarios but struggle to recognize categories beyond those seen during training. Open-vocabulary object detection (OVD) addresses this limitation by coupling visual features with natural language cues. Through large-scale multi-modal pretraining, images and text are aligned in a shared embedding space, enabling detectors to localize and classify objects described by text prompts.

Early OVD systems distilled knowledge from frozen CLIP encoders into two-stage detectors (Fukui et al., 2019). For example, ViLD (Gu et al., 2022) demonstrated that Mask R-CNN (He et al., 2018) trained with vision-language distillation can localize novel LVIS categories (Gupta et al., 2019). GLIP (Li et al., 2022) advanced this idea by jointly training phrase grounding and detection on 27M image-text pairs, producing representations that natively capture region-language alignment. Transformer-based models subsequently built on this foundation: OWL-ViT (Minderer et al., 2022) pre-trains a vanilla ViT (Dosovitskiy et al., 2021) and fine-tunes it end-to-end for open-world localization, reaching performance on par with task-specific detectors. OV-DETR (Zang et al., 2022) incorporates conditional matching to handle arbitrary queries, and Grounding DINO (Liu et al., 2024) introduces a cross-modality decoder that integrates text and visual cues, achieving substantial gains in open-vocabulary localization. MQ-Det (Xu et al., 2023) extends GLIP with a gated adapter that accepts both text and image queries, and OWL-ViT (Minderer et al., 2022) can also be adapted to support image-guided detection.

### 2.2 Few-shot adaptation of vision language models

Although foundation VLMs exhibit strong generalization, adapting them to domain-specific tasks using a supervised fine-tuning approach is often challenging due to the scarcity of labeled data. Madan et al. (2025) showed that, under a stricter K-shot evaluation protocol aligned to application semantics, powerful open-vocabulary localizers such as Grounding DINO can already surpass several classical few-shot detectors without fine-tuning. This highlights the strength of pretrained cross-modal alignment for localization under sparse supervision. Furthermore, Han and Lim (2024) proposed FM-FSOD, which leverages modern vision foundation models such as DINOv2 (Oquab et al., 2024) as the visual backbone and employs an LLM to reason over query image proposals using contextualized few-shot support images. This approach outperformed conventional learned heads on VOC/COCO while requiring minimal task-specific tuning, suggesting that textual reasoning can substitute for heavy detector training when labeled examples are scarce.

Instance-level personalization has also been explored in IPLoc (Doveh et al., 2025), which fine-tunes a VLM with dialogue-style supervision on a small set of annotated images, each containing a category label and bounding box. The model is then tasked with localizing the same object type in a query image. This approach enables reliable localization of user-specified objects while preserving general capabilities, providing a practical pathway for on-the-fly, user-guided adaptation when target appearances diverge from generic class descriptions. These approaches demonstrate the versatility of VLMs with few-shot samples, but they have primarily been evaluated on natural images and general-purpose domains. Their effectiveness in specialized settings such as microscopy, however, remains unclear. To address this gap, our study focuses on the microscopy domain. It analyzes when in-context examples are most effective, how many in-context few-shot support samples are needed, and how they should be allocated between localization and classification under severe domain shift.

### 2.3 Few-shot detection in biomedical and microscopic images

Microscopic imagery poses unique challenges, such as objects of interest that are often small, densely packed, low in contrast, and subject to modality-specific artifacts, which frequently degrade the performance of detectors trained on natural images. Rade et al. (2022) trained YOLOv3 (Redmon and Farhadi, 2018) using 114 annotated phase-contrast images to localize round, spindle, and polygonal live-cell morphologies, enabling automated region selection for downstream analysis. Early transformer-based efforts such as Cell-DETR (Prangemeier et al., 2020) adapted DETR (Carion et al., 2020) for yeast-cell instance segmentation and detection, demonstrating the feasibility of set prediction in biomedical settings. Cell DINO (Liao et al., 2024) extended Mask DINO (Li et al., 2023) into an end-to-end Transformer-based framework for joint cell segmentation and tracking. However, it operates as a closed-set detector without language conditioning, producing segmentation masks, bounding boxes, and tracking parameters for all detected cells. More recently, FSDAOD (Inayat et al., 2024) addressed cross-domain adaptation with only a few target shots, achieving state-of-the-art performance on two public cell datasets through class sampling and inter/intra-domain feature alignment. Verma et al. (2024) evaluated the performance of several VLMs, including GPT, LLaVA (Liu et al., 2023), and Gemini, as well as the Segment Anything Model (SAM) (Kirillov et al., 2023), on various tasks (classification, segmentation, counting, and visual-question answering) involving microscopy images. They found that while models like GPT and Gemini show promise in understanding visual features in microscopy images, their performance is not yet comparable to that of a human domain expert. They are easily challenged by common complexities in these images, such as impurities, defects, and overlapping artifacts. To provide a standardized benchmark, μ-Bench (Lozano et al., 2024) compiled 22 biomedical perception tasks, including object detection, across electron, fluorescence, and light microscopy, and showed that current VLMs struggle even on biologically simple cases such as images containing only the target object, where basic foreground-background segmentation would generally suffice. Complementing this, (Robicheaux et al., 2025) introduced Roboflow100-VL, a large-scale benchmark highlighting the poor generalization of VLMs to out-of-distribution domains, particularly in medical imaging under zero-shot and few-shot conditions.

Although VLMs can be adapted with only a few examples, their robustness in microscopic imaging remains limited. In this work, we address this gap by applying few-shot VLM adaptation to diverse microscopy images spanning different biological cell types and by systematically evaluating the object detection task.

## 3 FSOD benchmark for optical microscopy

We introduce Micro-OD, a benchmark designed to evaluate Vision-Language Models for few-shot cell detection and classification in microscopy. Unlike prior benchmarks that sample broadly from dissimilar classes (Bennequin et al., 2022), Micro-OD targets fine-grained visual localization where cell types share high semantic and morphological similarity. The benchmark integrates three public datasets along with an in-lab curated dataset, spanning diverse imaging modalities and experimental conditions. By providing a curated, multi-source dataset with dedicated few-shot splits, Micro-OD helps in an evaluation framework that addresses the lack of standardized resources for few-shot evaluation in medical imaging.

### 3.1 Datasets

**Blood Cell Count Dataset (BCCD)** (Shenggan, 2017): A publicly available dataset with 364 bright-field microscopy images annotated as the three blood cell categories of Platelets, Red Blood Cells (RBCs), and White Blood Cells (WBCs). This dataset is intensely used as a benchmark for cell detection and has bounding-box level annotations for ground-truth evaluation.

**Malaria (Plasmodium Vivax) (Broad Bioimage Benchmark Collection - BBBC) Dataset** (Ljosa et al., 2012): A publicly available collection that has 1,328 bright-field microscopy images of thin blood smears annotated into multiple parasitic stages (e.g., Gametocyte, Ring, Schizont, Trophozoite; with RBCs and WBCs). This dataset captures the morphological variation of cells and serves as a good testbed for characterizing fine-grained cell classification.

**Rat C6 (LIVECell subset) Dataset** (Edlund et al., 2021): A publicly available phase-contrast microscopy dataset with 456 images of the rat C6 glioma cell line. The original LIVECell dataset is unguided in shape-based annotations, so we re-annotated the images into three morphological categories: Polygonal, Round, and Spindle cells. Because C6 cells exhibit irregular and heterogeneous morphologies, this dataset is useful for testing shape-based generalization.

**NIH 3T3 Dataset (BACM lab collection, Iowa State University)**: Beyond publicly available sources, we also put together a small in-lab collection of 63 phase-contrast microscopy images of the NIH 3T3 fibroblast cell line. These microscopic images are annotated using the same morphology-based object detection categories as applied to Rat-C6 cells. These images are collected using the camera system attached to an atomic force microscopy (AFM) platform. The AFM employed two cameras: a top camera (640 × 480 resolution) and a bottom camera (1388 × 1040 resolution), as well as an additional level of optical zoom at 20×.

### 3.2 Micro-OD benchmark

Curating the dataset, we sample 63 slides from each source dataset with the largest number of annotated boxes, creating a pool of 252 candidate images. We divide this pool into two disjoint splits—“test” and “example”—using a two-phase Integer Linear Programme (ILP). For each source dataset, we select 53 test slides and 10 example slides, as summarized in Table 1.

**Table 1:** Test and example split counts for the Micro-OD benchmark.

BCCD (total slides: 364 images; slides split: 53 Test, 10 Example)		
Class	Ref	Sup
Platelets	159	10
Red Blood Cells	737	58
White Blood Cells	56	10
BBBC (total slides: 1328 images; slides split: 53 Test, 10 Example)		
Class	Ref	Sup
Gametocyte Cells	24	6
Red Blood Cells	3690	684
Ring Cells	34	6
Schizont Cells	10	6
Trophozoite Cells	193	26
White Blood Cells	49	6
NIH-3T3 (total slides: 63 images; slides split: 53 Test, 10 Example)		
Class	Ref	Sup
Polygonal Cells	303	43
Round Cells	11	6
Spindle Cells	62	13
LIVECell (total slides: 420 images; slides split: 53 Test, 10 Example)		
Class	Ref	Sup
Polygonal Cells	114	15
Round Cells	13	6
Spindle Cells	96	19
**Total cells**	**5551**	**914**
**Total cell slides**	**212**	**40**

The table reports the number of annotated cells across the four datasets (BCCD, BBBC, NIH-3T3, LIVECell) and their respective test (Ref) and example (Sup) splits.

The ILP is solved using PuLP (v3.0.2) with the CBC backend, zero MIP gap tolerance, and no time limit; trials that do not return a certified optimal solution are discarded. Phase 1 maximizes class–image coverage in the support set, recording the optimal coverage Cov^⋆^. Phase 2 locks coverage at Cov^⋆^ and simultaneously minimizes surplus support boxes while maximizing the weighted box count in the reference set, using inverse-frequency class weights to avoid domination by frequent classes. Because CBC's branch-and-bound is sensitive to variable ordering, we repeat the two-phase solve across *T* = 1000 shuffled image orderings, each yielding a potentially distinct optimal solution. The split with the highest Support-Spread Score (SSS) is retained:


$$\begin{array}{l} \text{SSS}=\text{CPC}\times \text{CBE}, \end{array}$$ (1)

where Class-Presence Coverage (CPC) measures the average fraction of support images containing each class, and Class-Balance Entropy (CBE) is the normalized entropy of the class box distribution. Ties are broken by retaining the earliest seed. Our final split achieves an SSS of 0.74 (CPC = 0.83, CBE = 0.89), indicating both high diversity and good class balance in the few-shot examples.

## 4 Benchmark evaluation strategy

To systematically evaluate the proposed benchmark, we consider a diverse set of multimodal models, ranging from foundation detectors such as OWL-ViT (Minderer et al., 2022) and Grounding DINO (Liu et al., 2024) to proprietary large-scale VLMs including GPT-4 (Achiam et al., 2023), Gemini-2.5 (Comanici et al., 2025), Claude-3.7 (Kurokawa et al., 2024), and their respective variants. We designed four experiments with the aim of determining the capabilities of these models in localizing and classifying objects using different input-output configurations.

**Figure 2:**
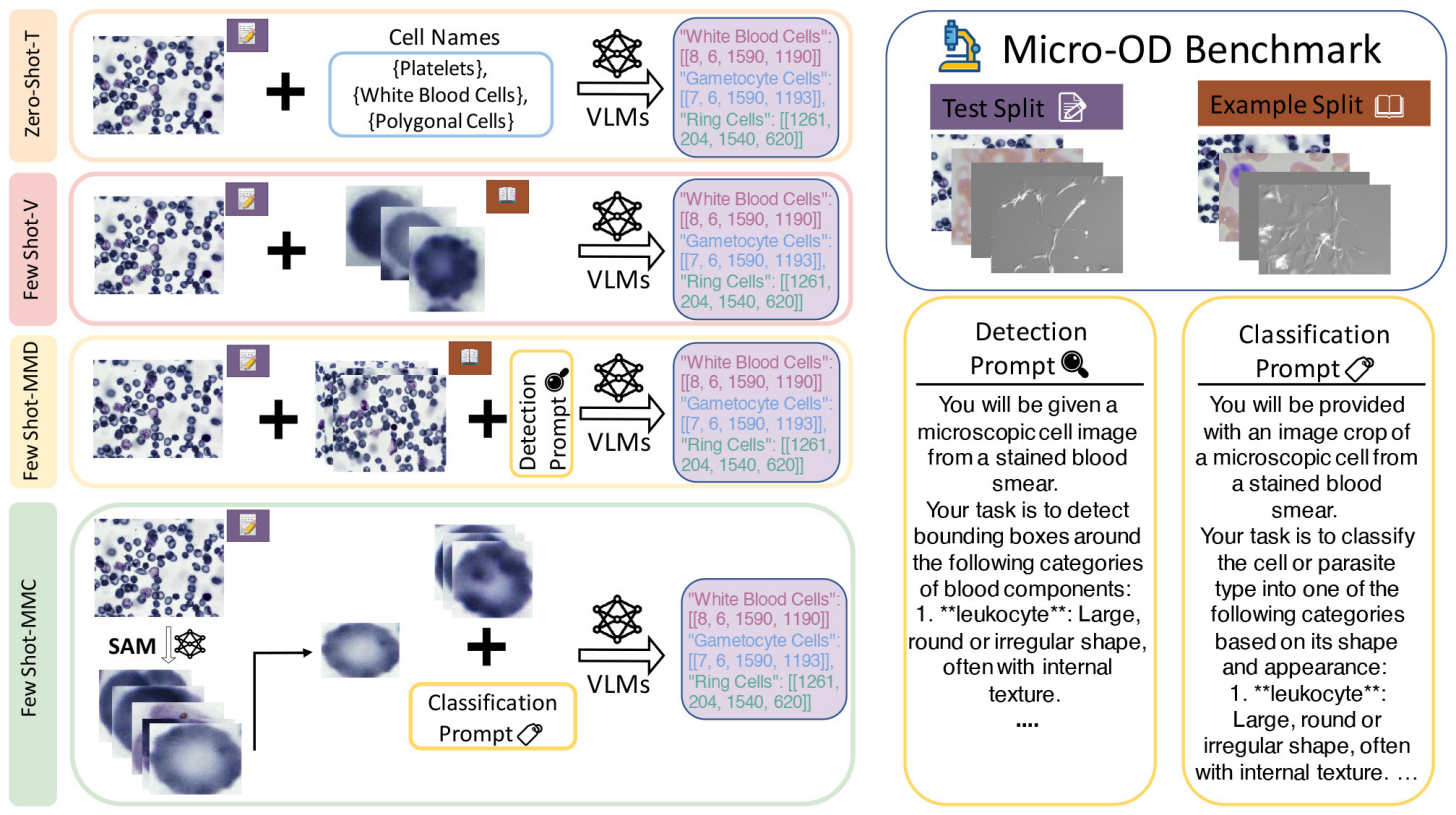
The four experimental configurations used to evaluate Vision-Language Models (VLMs) on the Micro-OD benchmark. This figure illustrates the distinct setups: Zero Shot-T (text-only prompt), Few Shot-V (visual-only prompts), Few Shot-MMO (multi-modal few-shot detection), and Few Shot-MMC (a cascaded pipeline of localization with SAM followed by few-shot classification with VLMs).

We outline the experimental planning methods below and provide descriptions of the four experimental configurations, as well as the role of few-shot adaptation in understanding model flexibility and robustness. We provide individual dataset-wise text prompt for applicable experiments in Section 1 of Supplementary material. Figure 2 provides an overview of the experimental configurations and their respective settings. The output consists of the bounding box coordinates for all experiments.

**1. Experiment Zero Shot-T**

**Input:** Text Prompt, Test Image

**Models:** OWL-ViT, Grounding DINO, Gemini-2.5-Flash, Gemini-2.5-Flash-Thinking, Claude-3.7-Sonnet, Claude-3.7-Sonnet-Thinking

In this baseline experimental condition, models were queried with pure text prompts that describe the target object classes, without visual examples (true zero-shot condition). This condition evaluated the models' open-vocabulary detection capabilities by asking them to predict bounding boxes and predictions based on class names without demonstration or fine-tuning context. The experiment was focused on zero-shot detection.

**2. Experiment Few Shot-V**

**Input:** Image Prompt, Test Image

**Models:** OWL-ViT

This experiment used OWL-ViT for detection using only visual context in a few-shot setting. In each instance, the model was presented with annotated example images containing both object crops and classes. In few-shot scenarios, 1, 3, or 6 object crops were provided with test image as example image prompts. This setup aimed to evaluate how well localization and classification task could be performed through visual demonstrations alone.

**3. Experiment Few-Shot-MMD**

**Input:** Text Prompt, Image Prompt, Test Image

**Models:** OWL-ViT, GPT-4o, GPT-5, Gemini-2.5-Flash, Gemini-2.5-Flash-Thinking, Claude-3.7-Sonnet, Claude-3.7-Sonnet-Thinking

In this experiment, we used state-of-the-art VLMs and tried to leverage ability for joint vision-language reasoning for object detection. For each detection task in API call, the model was given the full image and text prompt describing the object classes, and 1, 3, or 6 demonstration examples in the prompt (few-shot). This investigated the accuracy and reliability of bounding box predictions and class assignments are impacted by incremental multi-modal examples.

**4. Experiment Few-Shot-MMC**

**Input:** Text Prompt, Image Prompt, Test Image

**Models:** OWL-ViT, GPT-4o, GPT-5, Gemini-2.5-Flash, Gemini-2.5-Flash-Thinking, Claude-3.7-Sonnet, Claude-3.7-Sonnet-Thinking

In the final experiment, object localization was conducted with the SAM (Segment Anything Model) (Kirillov et al., 2023) to crop out object areas from the full images. The predicted cropped areas and classification prompt as text with 1, 3, or 6 demonstration examples were passed to the state-of-the-art generative VLMS for classification. This cascaded setup evaluated the models' ability to assign accurate class labels to object areas when provided with explicit textual demonstrations, while specifically evaluating the impact of few-shot text-based support.

### 4.1 Implementation and reproducibility details

To ensure reproducibility of the experiments reported in Table 2, we provide detailed implementation settings covering VLM inference parameters, detector configurations, and preprocessing steps. Unless otherwise noted, these settings were consistent across datasets and experimental trials.

**Table 2:** Evaluation results grouped by metric (mF1 and Mean IoU) across three conditions (K = 1,3,6).

Method	mF1 ↑			Mean IoU ↑		
	K = 1	K = 3	K = 6	K = 1	K = 3	K = 6
Few-Shot-V						
OWL-ViT	**0.08** **±0.0020**	**0.12** **±0.0212**	**0.17** **±0.0141**	**0.78** **±0.0141**	**0.80** **±0.0071**	**0.81** **±0.0010**
Few-Shot-MMD						
GPT-4o	0.05 ± 0.0021	0.06 ± 0.0148	0.07 ± 0.0290	0.50 ± 0.1131	0.54 ± 0.0778	0.53 ± 0.0424
GPT-5	0.07 ± 0.0010	0.08 ± 0.0014	0.09 ± 0.0087	0.48 ± 0.0247	0.44 ± 0.1665	0.57 ± 0.0325
Gemini-2.5-Flash	0.05 ± 0.0141	0.07 ± 0.0219	0.07 ± 0.0424	0.53 ± 0.0778	0.59 ± 0.0141	0.55 ± 0.0990
Gemini-2.5-Flash-thinking	**0.19** **±0.0283**	**0.22** **±0.0156**	**0.23** **±0.0212**	**0.76** **±0.0636**	**0.77** **±0.0707**	**0.77** **±0.0707**
Claude-3.7-Sonnet	0.04 ± 0.0141	0.04 ± 0.0177	0.05 ± 0.0226	0.17 ± 0.0495	0.17 ± 0.0778	0.44 ± 0.0120
Claude-3.7-Sonnet-thinking	0.05 ± 0.0141	0.06 ± 0.0212	0.07 ± 0.0233	0.42 ± 0.0424	0.39 ± 0.1344	0.47 ± 0.0636
Few-Shot-MMC						
GPT-4o	0.24 ± 0.0059	0.27 ± 0.0141	**0.29** **±0.0161**	**0.81** **±0.0276**	**0.81** **±0.0245**	**0.82** **±0.0348**
GPT-5	**0.26** **±0.0011**	**0.27** **±0.0003**	0.28 ± 0.0004	0.79 ± 0.0040	0.78 ± 0.0037	0.78 ± 0.0015
Gemini-2.5-flash	0.21 ± 0.0095	0.21 ± 0.0107	0.22 ± 0.0028	0.78 ± 0.0081	0.79 ± 0.0051	0.79 ± 0.0052
Gemini-2.5-flash-thinking	0.16 ± 0.0564	0.18 ± 0.0223	0.17 ± 0.0339	0.63 ± 0.0141	0.63 ± 0.0200	0.56 ± 0.0021
Claude-3.7-Sonnet	0.10 ± 0.0298	0.14 ± 0.0262	0.13 ± 0.0320	0.79 ± 0.0141	0.79 ± 0.0122	0.79 ± 0.0195
Claude-3.7-sonnet-thinking	0.09 ± 0.0303	0.15 ± 0.0303	0.16 ± 0.0401	0.78 ± 0.0078	0.79 ± 0.0124	0.79 ± 0.0144

Bold values indicate the best-performing result for each metric and setting.

#### 4.1.1 VLM API configuration

All VLMs were queried with temperature set to 0 across all models, top-*p* sampling set to 1.0, and top-*k* sampling not used. Each query generated a single response, and no explicit stop sequences were specified. While temperature 0 corresponds to greedy decoding in theory, in practice LLM inference via cloud APIs remains non-deterministic due to floating-point non-associativity under variable server load and batch sizes (He and Lab, 2025). Consequently, residual variance across trials arises from both few-shot example sampling and API-level inference nondeterminism. To isolate the former, three fixed random seeds (42, 123, and 456) were used for few-shot example sampling across the three trials, and results are reported as mean ± standard deviation.

For OpenAI models, we used GPT-4o (gpt-4o-2024-08-06), GPT-5 (gpt-5-2025-08-07), and GPT-o4-mini (version unspecified). For Google models, we used Gemini-2.5-Flash with a maximum output length of 4096 tokens. For Anthropic models, we used Claude-3.7-Sonnet (claude-3-7-sonnet-20250219) with a maximum output length of 8096 tokens. Reasoning (“thinking”) variants were enabled where applicable using provider-specific controls. For Gemini-2.5-Flash-Thinking, reasoning was enabled with an unlimited token budget (budget = −1). For Claude-3.7-Sonnet-Thinking, extended thinking mode was enabled with a reasoning budget of 3096 tokens. Non-thinking variants were run with reasoning disabled. No explicit reasoning controls were used for OpenAI models.

#### 4.1.2 Detector settings

For open-vocabulary detection experiments using OWL-ViT, we used the google/owlvit-base-patch32 checkpoint.^1^ The score threshold was set to 0.05, and non-maximum suppression (NMS) was applied with an IoU threshold of 0.50. No explicit limit was imposed on the maximum number of detections per image.

#### 4.1.3 SAM configuration

For the cascaded localization pipeline (Few-Shot-MMC), we used the Segment Anything Model (SAM) (Kirillov et al., 2023) with the ViT-H backbone and checkpoint sam_vit_h_4b8939.pth, operated in automatic mask generation mode to produce candidate (*x*_1_, *y*_1_, *x*_2_, *y*_2_) and expanded with a padding of 10 pixels, clipped to image boundaries. Bounding boxes were precomputed offline and cached to ensure consistent proposals across trials. The generated bounding boxes were used to crop image regions, which were then resized to 128 × 128 pixels using bilinear interpolation.

## 5 Results

We conduct the discussed experiments with state-of-the-art Vision-Language models on our microscopic benchmark and report the overall result in this section.

### 5.1 Metrics

In microscopic images, detection performance is influenced by two distinct factors: (i) whether the model can reliably find relevant cell instances across diverse backgrounds and modalities, and (ii) whether the predicted bounding boxes tightly localize the detected cells. To build intuition for the experimental results, we report metrics that disentangle these two aspects: mF1 summarizes detection quality across multiple localization strictness levels, while Mean IoU (TP@0.5) quantifies the spatial alignment of detections that are already deemed correct at a standard operating point.

Algorithm 1 Few-shot dataset split via two-phase ILP. 
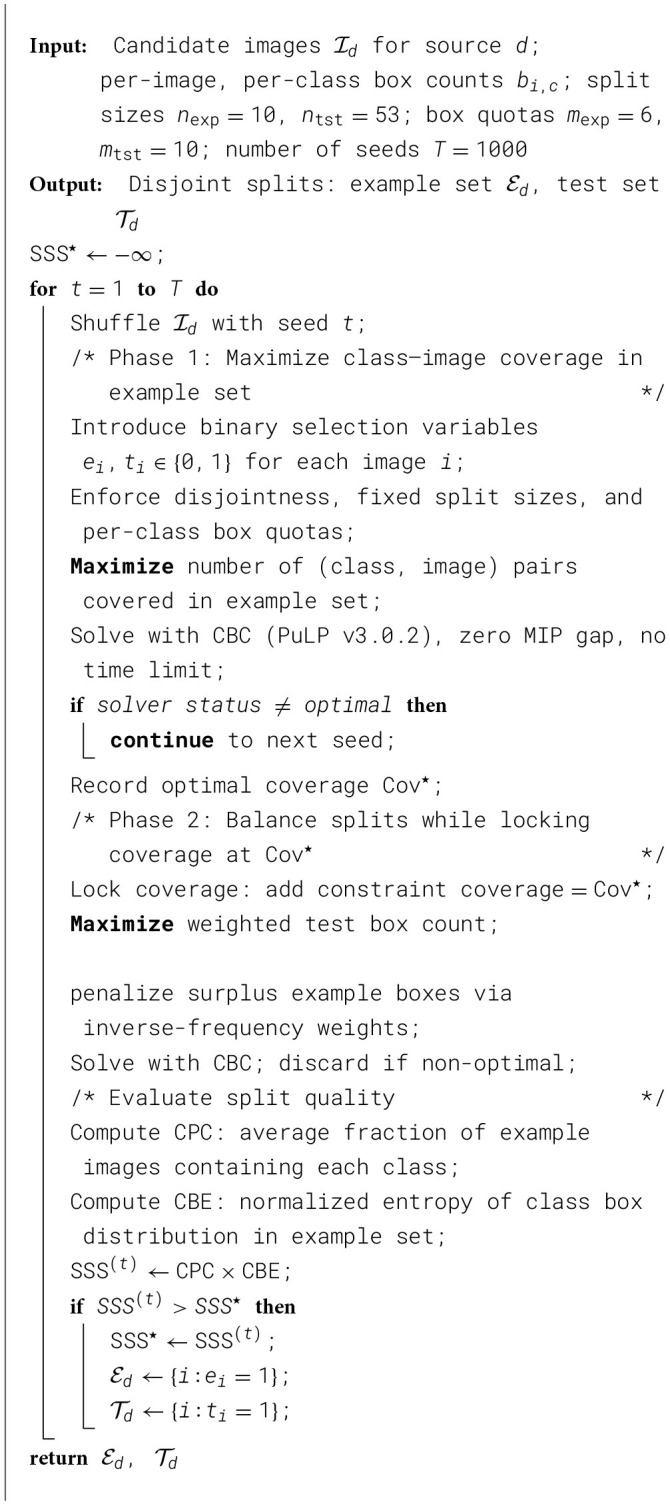

To compute these metrics, we first match predicted bounding boxes to ground truth boxes using the Hungarian algorithm, inspired by DETR (Carion et al., 2020). We chose this approach over a greedy matching strategy because microscopy images can contain many densely packed instances, where duplicate predictions or clustered false positives can inflate performance under greedy matching. One-to-one optimal matching provides a consistent basis for defining true positives, false positives, and false negatives prior to computing precision and recall.

We then calculate precision and recall at each of 50 distinct Intersection over Union (*IoU*) thresholds, ranging from 0.05 to 0.70. The F1 score for a given threshold is the harmonic mean of precision and recall:


$$\begin{array}{l} F1=\frac{2\times \text{Precision}\times \text{Recall}}{\text{Precision}+\text{Recall}} \end{array}$$ (2)

The final *mF*1 is the mean of the F1 scores obtained across these 50 *IoU* thresholds:


$$\begin{array}{l} mF1=\frac{\sum_{IoU=0.05}^{0.70}F1@IoU}{50} \end{array}$$ (3)

We report this aggregated score because it evaluates detection robustness across varying localization criteria. In particular, lower IoU thresholds primarily test whether the model can place boxes on the correct objects (coarse localization), whereas higher thresholds increasingly require tight spatial overlap. As a result, mF1 reflects a model's overall reliability: high mF1 requires simultaneously achieving good recall (covering most true instances) and good precision (avoiding spurious detections) across a spectrum of overlap strictness, which is well-suited to microscopy where objects are small, heterogeneous, and often affected by imaging artifacts.

In addition to *mF*1, we also report the Mean IoU of true positives at *IoU* = 0.5 (Mean IoU (TP@0.5)). This metric captures the average overlap of all predicted boxes that were successfully matched to a ground truth instance at the *IoU* = 0.5 threshold, which is a standard operating point in object detection benchmarks. Formally, for each true positive match at *IoU* ≥ 0.5, we compute its IoU with the ground truth box; the final score is the arithmetic mean across all such matches. If no true positives are found at this threshold, the metric is defined as 0. This measure complements mF1 by focusing specifically on localization accuracy conditional on correctness. Importantly, because Mean IoU (TP@0.5) is computed only over successful matches, it does not directly penalize missed instances or false positives. Consequently, it is possible for a model to achieve a relatively high Mean IoU while still exhibiting a low mF1 if it produces only a small number of correct detections (e.g., low recall), but those few detections align well with ground truth. Conversely, a model may achieve moderate mF1 while having lower Mean IoU if it detects many instances but with loose or imprecise boxes. Reporting both metrics therefore helps distinguish “coverage” failures from “tight localization” failures in the microscopy domain.

### 5.2 Zero-shot detection

Under true zero-shot conditions, where vision foundation detectors were given only text-based prompts, the models were unable to generate bounding boxes of good fit. This reflects the significant domain gap between their natural-image pre-training and the specialized characteristics of microscopy. We evaluate the zero-shot performance of six Vision Language Models and report their scores in Table 3. Grounding DINO achieved an *mF*1 score of 0.04 with a Mean *IoU* of 0.53, while OWL-ViT scored an *mF*1 of 0.03 with a Mean *IoU* of 0.40. Among the new models, Gemini-2.5-Flash and its thinking variant performed better, with the latter achieving *mF*1 of 0.06 and Mean IoU of 0.59. In contrast, Claude-3.7-Sonnet and its thinking variant showed lower performance, scoring *mF*1 0.03. These results indicate that while the detectors can occasionally place bounding boxes with reasonable overlap (as shown by the moderate Mean *IoU*), their overall detection quality across a range of *IoU* thresholds is low without visual examples.

**Table 3:** Evaluation results for few-shot object detection in the zero-shot setting.

Method	mF1 [0.05: 0.70] ↑	Mean IoU (TP@0.5) ↑
EXP1: zero shot-T		
GroundingDINO	0.04	0.53
OWL-ViT	0.03	0.40
Gemini-2.5-Flash	0.05	0.57
Gemini-2.5-Flash-Thinking	**0.06**	**0.59**
Claude-3.7-Sonnet	0.03	0.34
Claude-3.7-Sonnet-Thinking	0.03	0.31

We report the mF1 score and Mean IoU (TP@0.5) for various VLMs. Bold values indicate the best-performing result for each metric and setting.

### 5.3 Few-shot detection

We next evaluate some of the leading large vision and mutlimodal models on this task, providing *K* = 1, 3, and 6 examples for in-context learning. For this evaluation, we report results as mean ± standard deviation over three trials to consider the variability introduced by few-shot demonstration sampling. Paired t-tests confirming the statistical significance of K=1 vs K=6 improvements for each model are provided in the Supplementary Table S10. We used a minimal temperature while generative sampling from the models to reduce the variability to the most extent. We report the overall results in Table 2 and granular dataset-wise results in section 2 of Supplementary Table S3–S9. As a conventional lightweight detector baseline under minimal supervision, YOLO11n in the K=6 few-shot setting achieved consistently low mF1 scores across datasets, indicating weak generalization in the low-data regime. This further motivates the investigation of VLM-based pipelines as a potential option for few-shot object detection. Detailed results are reported in Supplementary Table S1.

**Experiment few shot-V**: Providing OWL-ViT with only visual examples (cell crops) yielded consistent improvements. As the number of shots increased from 1 to 6, the mF1 score increased from 0.08 to 0.17, while the Mean IoU(TP@0.5) remained high in the range of 0.78 to 0.81. The relatively high Mean IoU suggests that when OWL-ViT produces correct matches, those detections tend to be reasonably well-aligned. However, the lower mF1 implies that many instances are still missed and/or predictions remain inconsistent under stricter overlap criteria.

**Experiment few shot-MMD**: In the multimodal few-shot detection setting (Few Shot-MMD), overall gains from adding multimodal demonstrations were strongly model dependent. Gemini-2.5-Flash-Thinking achieved the best performance and benefited consistently from additional examples, improving from 0.19 to 0.23, while maintaining high localization quality with Mean IoU (TP@0.5) of nearly 0.76. In contrast, the performance of the non-thinking counterpart was not at par with mF1 of 0.07 after 6 examples. Multimodal few-shot prompting can improve end-to-end localization, but the advantage is concentrated in specific models, notably Gemini-2.5-Thinking.

**Experiment few shot-MMC**: In this setting, where SAM provides object crops and the VLM is responsible primarily for fine-grained classification, overall performance improved substantially relative to Few Shot-MMD and exhibited a different ranking of model families. The GPT-family models were consistently strong and stable: GPT-4o increased from 0.24 to 0.29 across the shots, with Mean IoU 0.81. In contrast to the Few Shot-MMD evaluation, Gemini-2.5-Flash-Thinking performed achieved relatievly lower scores with mF1 upto 0.17 and Mean IoU upto 0.56.

Taken together, few-shot support improves performance over the zero-shot baseline, but results are strongly configuration dependent. End-to-end multimodal detection (Few Shot-MMD) remains challenging and yields limited gains for several models, whereas the cascaded pipeline (Few Shot-MMC) produces substantially higher and more stable performance by decoupling localization from classification.

## 6 Discussion

Our study highlights both the potential and the limitations of adapting vision-language models to the microscopic imaging domain. The results from our comprehensive evaluation on the Micro-OD benchmark yield several vital observations.

The performance gap between zero-shot and few-shot settings illustrates the critical role of in-context adaptation. While zero-shot VLMs consistently struggled due to the domain shift from natural images to microscopy, the benefit of one-shot support was limited. Most models showed little or no gain at a few shots, with the exception of the Gemini-2.5-Flash-Thinking variant, which demonstrated clear improvements. This suggests that a minimal number of in-context examples may be sufficient to anchor prior knowledge in the target domain, but the effect depends strongly on the model architecture.

In this context, the effect of “thinking” variants can be interpreted as an empirical observation rather than a causal mechanism. While Gemini-2.5-Flash-Thinking shows notable gains in end-to-end detection (Few-Shot-MMD), this trend is not consistent across all thinking models (e.g., Claude-3.7-Sonnet-Thinking), indicating that multi-modal reasoning modes are not uniform across providers. Moreover, the relative advantage differs across tasks: gains are more evident in detection, whereas non-thinking variants are often stronger in the cascaded classification setting (Few-Shot-MMC). One possible explanation is that thinking modes implicitly increase test-time computation, but the benefit appears model- and task-dependent.

Although the FSOD pipeline achieves an mF1 of about 0.30, representing a fivefold improvement over the zero-shot baseline, its overall reliability remains limited given the still-low absolute mF1 score. Also, each dataset in Micro-OD contains only 53 test images, so aggregate metrics can be sensitive to a small number of difficult cases. The main limitations in terms of performance lie in the following three aspects specific to microscopic images that were not encountered during the initial pre-training: high cell density, violating the assumption of spatial independence; heterogeneous background noise, such as debris and uneven staining; and intrinsic differences in pixel statistics and object sizes between the two domains.

## 7 Conclusions

We present the Micro-OD benchmark and a systematic evaluation of state-of-the-art vision-language models for few-shot object detection in microscopic imagery. Our experiments demonstrate that VLMs can be adapted for improved performance in this specialized biomedical domain with only a few annotated examples, improving over zero-shot baselines. However, their effectiveness is strongly conditioned on the task design and underlying model architecture. Our analysis revealed a task-dependent effect of implicit reasoning mechanisms, where “thinking” tokens are more effective for complex end-to-end localization, while simpler, non-thinking models excel at fine-grained classification of pre-localized objects. Future extensions of the benchmark can include a broader range of cell types and incorporate diverse imaging modalities and acquisition settings to evaluate generalization. The Micro-OD benchmark provides a standardized foundation for these future studies, paving the way for the development of more capable AI assistants for biomedical imaging.

## Data Availability

The dataset analyzed for this study can be found in: https://huggingface.co/datasets/stumbledparams/Micro-OD.

## Footnotes

1
https://huggingface.co/google/owlvit-base-patch32

## References

[B1] Achiam J. Adler S. Agarwal S. Ahmad L. Akkaya I. Aleman F. L. . (2023). GPT-4 technical report. arXiv [preprint] arXiv:2303.08774. doi: 10.48550/arXiv.2303.08774

[B2] Bennequin E. Tami M. Toubhans A. Hudelot C. (2022). “Few-shot image classification benchmarks are too far from reality: build back better with semantic task sampling,” in Proceedings of the IEEE/CVF Conference on Computer Vision and Pattern Recognition (CVPR) Workshops (New Orleans, LA: IEEE), 4767–4776.

[B3] Caicedo J. C. Cooper S. Heigwer F. Warchal S. Qiu P. Molnar C. . (2017). Data-analysis strategies for image-based cell profiling. Nat. Methods 14, 849–863. doi: 10.1038/nmeth.4397 28858338 PMC6871000

[B4] Carion N. Massa F. Synnaeve G. Usunier N. Kirillov A. Zagoruyko S. (2020). “End-to-end object detection with transformers,” in Computer Vision – *ECCV 2020: 16th European Conference* (Berlin, Heidelberg: Springer-Verlag), 213–229.

[B5] Comanici G. Bieber E. Schaekermann M. Pasupat I. Sachdeva N. Dhillon I. . (2025). Gemini 2.5: Pushing the frontier with advanced reasoning, multimodality, long context, and next generation agentic capabilities. arXiv [preprint] arXiv:2507.06261. doi: 10.48550/arXiv.2507.06261

[B6] Dosovitskiy A. Beyer L. Kolesnikov A. Weissenborn D. Zhai X. Unterthiner T. . (2021). An image is worth 16x16 words : transformers for image recognition at scale. arXiv [Preprint].

[B7] Doveh S. Shabtay N. Lin W. Schwartz E. Kuehne H. Giryes R. . (2025). Teaching VLMs to localize specific objects from In-context examples. arXiv [preprint] arXiv:2411.13317. doi: 10.48550/arXiv.2411.13317

[B8] Edlund C. Jackson T. R. Khalid N. Bevan N. Dale T. Dengel A. . (2021). Livecell–a large-scale dataset for label-free live cell segmentation. Nat. Methods 18, 1038–1045. doi: 10.1038/s41592-021-01249-6 34462594 PMC8440198

[B9] Fukui S. Yu J. Hashimoto M. (2019). “Distilling knowledge for non-neural networks,” in 2019 Asia-Pacific Signal and Information Processing Association Annual Summit and Conference (APSIPA ASC) (Piscataway, NJ: IEEE), 1411–1416.

[B10] Gu X. Lin T.-Y. Kuo W. Cui Y. (2022). Open-vocabulary object detection via vision and language knowledge distillation. arXiv [preprint] arXiv:2104.13921. doi: 10.48550/arXiv.2104.13921

[B11] Gupta A. Dollar P. Girshick R. (2019). “LVIS: a dataset for large vocabulary instance segmentation,” in 2019 IEEE/CVF Conference on Computer Vision and Pattern Recognition (CVPR) (Los Alamitos, CA: IEEE Computer Society), 5351–5359.

[B12] Gurcan M. N. Boucheron L. E. Can A. Madabhushi A. Rajpoot N. M. Yener B. (2009). Histopathological image analysis: a review. IEEE Rev. Biomed. Eng. 2, 147–171. doi: 10.1109/RBME.2009.2034865 20671804 PMC2910932

[B13] Han G. Lim S.-N. (2024). “Few-shot object detection with foundation models,” in Proceedings of the IEEE/CVF Conference on Computer Vision and Pattern Recognition (CVPR) (Seattle: IEEE), 28608–28618.

[B14] He H. Lab T. M. (2025). “Defeating nondeterminism in LLM inference,” in Thinking Machines Lab: Connectionism.

[B15] He K. Gkioxari G. Dollár P. Girshick R. (2018). Mask R-CNN. arXiv [Preprint]. 29994331 10.1109/TPAMI.2018.2844175

[B16] Inayat S. Dilawar N. Sultani W. Ali M. (2024). “Few-shot domain adaptive object detection for microscopic images,” in Proceedings of Medical Image Computing and Computer Assisted Intervention-MICCAI 2024 (Cham: Springer Nature), 98–108.

[B17] Kirillov A. Mintun E. Ravi N. Mao H. Rolland C. Gustafson L. . (2023). “Segment anything,” in Proceedings of the IEEE/CVF International Conference on Computer Vision, pages (Los Alamitos, CA: IEEE Computer Society), 4015–4026.

[B18] Kurokawa R. Ohizumi Y. Kanzawa J. Kurokawa M. Sonoda Y. Nakamura Y. . (2024). Diagnostic performances of Claude 3 Opus and Claude 3.5 Sonnet from patient history and key images in radiology's 'Diagnosis Please' cases. Jpn. J. Radiol. 42, 1399–1402. doi: 10.1007/s11604-024-01634-z 39096483 PMC11588754

[B19] Li F. Zhang H. Xu H. Liu S. Zhang L. Ni L. M. . (2023). “Mask DINO: towards a unified transformer-based framework for object detection and segmentation,” in Proceedings of the IEEE/CVF Conference on Computer Vision and Pattern Recognition (Los Alamitos, CA: IEEE Computer Society), 3041–3050. doi: 10.1109/CVPR52729.2023.00297

[B20] Li L. H. Zhang P. Zhang H. Yang J. Li C. Zhong Y. . (2022). “Grounded language-image pre-training,” in 2022 IEEE/CVF Conference on Computer Vision and Pattern Recognition (CVPR) (Los Alamitos: IEEE Computer Society), 10955–10965.

[B21] Liao W. Luo L. Wang C. Zhang C. (2024). “Cell DINO: end-to-end cell segmentation and tracking with transformer,” in 2024 IEEE International Conference on Bioinformatics and Biomedicine (BIBM) (Lisbon: IEEE), 3491–3494.

[B22] Litjens G. Kooi T. Bejnordi B. E. Setio A. A. A. Ciompi F. Ghafoorian M. . (2017). A survey on deep learning in medical image analysis. Med. Image Anal. 42, 60–88. doi: 10.1016/j.media.2017.07.005 28778026

[B23] Liu H. Li C. Wu Q. Lee Y. J. (2023). Visual instruction tuning. arXiv [Preprint].

[B24] Liu S. Zeng Z. Ren T. Li F. Zhang H. Yang J. . (2024). “Grounding DINO: marrying DINO with grounded pre-training for open-set object detection,” in European Conference on Computer Vision (Cham: Springer), 38–55.

[B25] Ljosa V. Sokolnicki K. L. Carpenter A. E. (2012). Annotated high-throughput microscopy image sets for validation. Nat. Methods 9, 637–637. doi: 10.1038/nmeth.2083 22743765 PMC3627348

[B26] Lozano A. Nirschl J. Burgess J. Gupte S. R. Zhang Y. Unell A. . (2024). “Micro-bench: a vision-language benchmark for microscopy understanding,” in Proceedings of the 38th International Conference on Neural Information Processing Systems, NIPS '24 (Red Hook, NY: Curran Associates Inc.), 965:1–965:15.

[B27] Madan A. Peri N. Kong S. Ramanan D. (2025). “Revisiting few-shot object detection with vision-language models,” in Proceedings of the 38th International Conference on Neural Information Processing Systems, NIPS '24 (Red Hook, NY: Curran Associates Inc.), 617:1–617:14.

[B28] Minderer M. Gritsenko A. Stone A. Neumann M. Weissenborn D. Dosovitskiy A. . (2022). “Simple open-vocabulary object detection,” in European Conference on Computer Vision (Cham: Springer), 728–755.

[B29] Oquab M. Darcet T. Moutakanni T. Vo H. V. Szafraniec M. Khalidov V. . (2024). “DINOv2: Learning robust visual features without supervision,” in Transactions on Machine Learning Research. Featured Certification.

[B30] Prangemeier T. Reich C. Koeppl H. (2020). “Attention-based transformers for instance segmentation of cells in microstructures,” in 2020 IEEE International Conference on Bioinformatics and Biomedicine (BIBM) (Seoul: IEEE), 2275–2282.

[B31] Rade J. Zhang J. Sarkar S. Krishnamurthy A. Ren J. Sarkar A. (2022). Deep learning for live cell shape detection and automated AFM navigation. Bioengineering 9:10. doi: 10.3390/bioengineering9100522 PMC9598706 36290490

[B32] Redmon J. Farhadi A. (2018). YOLOv3: an incremental improvement. arXiv [Preprint].

[B33] Ren S. He K. Girshick R. Sun J. (2015). “Faster R-CNN: towards real-time object detection with region proposal networks,” in Proceedings of the 29th International Conference on Neural Information Processing Systems - *Volume 1, NIPS'15* (Cambridge, MA: MIT Press), 91–99.

[B34] Robicheaux P. Popov M. Madan A. Robinson I. Nelson J. Ramanan D. . (2025). Roboflow100 -VL: a multi-domain object detection benchmark for vision-language models. arXiv [Preprint].

[B35] Shen D. Wu G. Suk H.-I. (2017). Deep learning in medical image analysis. Annu. Rev. Biomed. Eng. 19, 221–248. doi: 10.1146/annurev-bioeng-071516-044442 28301734 PMC5479722

[B36] Shenggan (2017). BCCD: blood cell count and detection dataset. GitHub Repository.

[B37] Smith A. S. Ankam S. Farhy C. Fiengo L. Basa R. C. Gordon K. L. . (2022). High-content analysis and kinetic image cytometry identify toxicity and epigenetic effects of HIV antiretrovirals on human iPSC-neurons and primary neural precursor cells. J. Pharmacol. Toxicol. Methods 114:107157. doi: 10.1016/j.vascn.2022.107157 35143957 PMC9103414

[B38] Suzuki K. (2017). Overview of deep learning in medical imaging. Radiol. Phys. Technol. 10:257–273. doi: 10.1007/s12194-017-0406-5 28689314

[B39] Verma P. Van M.-H. Wu X. (2024). “Beyond Human Vision: The Role of Large Vision Language Models in Microscope Image Analysis,” in 2024 IEEE International Conference on Big Data (BigData) (Los Alamitos, CA: IEEE Computer Society), 1700–1705.

[B40] Weissleder R. Nahrendorf M. (2015). Advancing biomedical imaging. Proc. Nat. Acad. Sci. 112, 14424–14428. doi: 10.1073/pnas.1508524112 26598657 PMC4664297

[B41] Xu Y. Zhang M. Fu C. Chen P. Yang X. Li K. . (2023). “Multi-modal queried object detection in the wild,” in Advances in Neural Information Processing Systems, eds. A. Oh, T. Naumann, A. Globerson, K. Saenko, M. Hardt, and S. Levine (New York: Curran Associates, Inc.), 4452–4469.

[B42] Zang Y. Li W. Zhou K. Huang C. Loy C. C. (2022). “Open-vocabulary DETR with conditional matching,” in Computer Vision – *ECCV 2022* (Cham: Springer Nature), 106–122.

[B43] Zhang J. Huang J. Jin S. Lu S. (2024). Vision-language models for vision tasks: a survey. IEEE Trans. Pattern Anal. Mach. Intell. 46, 5625–5644. doi: 10.1109/TPAMI.2024.3369699 38408000

[B44] Zhou H. Nova A. Larochelle H. Courville A. Neyshabur B. Sedghi H. (2022). Teaching algorithmic reasoning via in-context learning. arXiv preprint arXiv:2211.09066. doi: 10.48550/arXiv.2211.09066

[B45] Zhou S. K. Greenspan H. Davatzikos C. Duncan J. S. Van Ginneken B. Madabhushi A. . (2021). A review of deep learning in medical imaging: imaging traits, technology trends, case studies with progress highlights, and future promises. Proc. IEEE 109, 820–838. doi: 10.1109/JPROC.2021.3054390 37786449 PMC10544772

[B46] Zou Z. Chen K. Shi Z. Guo Y. Ye J. (2023). Object detection in 20 years: a survey. Proc. IEEE 111, 257–276. doi: 10.1109/JPROC.2023.3238524

