# Supplementary material for: In-context adaptation of VLMs for few-shot cell detection in optical microscopy
Source: Front Artif Intell. 2026 May 15;9:1761903. doi: 10.3389/frai.2026.1761903 (PMC13219282; doi:10.3389/frai.2026.1761903)
Supplement: Supplementary file 1 [file Data_Sheet_1.pdf]

Supplementary Material

1 CELL DETECTION PROMPTS

Listing 1. Detection prompt based on cell-name for benchmarking on BCCD dataset

```
################################################
# Detection Prompt for BCCD dataset #
################################################

You will be given a microscopic RGB image of a human blood smear. Your task
   is to detect and generate bounding boxes around the following three
   categories of blood components:

1. **Platelets**  Small, irregularly shaped fragments. They lack a nucleus
   and appear as tiny, scattered specks.
2. **Red Blood Cells (RBCs)**  Round, biconcave discs with a smooth,
   circular outline. They often appear lighter in the center.
3. **White Blood Cells (WBCs)**  Larger cells with a distinct, often multi-
   lobed nucleus. Their cytoplasm may contain visible granules.

Return **only** a JSON object with exactly these three keys: `"Platelets"`,
   `"RBC"`, and `"WBC"`. Each key must map to a list of bounding boxes, with
    each bounding box defined as an array of four integers: `[x1, y1, x2, y2
   ]` (pixel coordinatriaes, where (x1, y1) is the top-left corner and (x2,
   y2) is the bottom-right corner).

**Important**:
- Return ONLY the bounding box coordinate directly
- You are not allowed to return other suggestions or code to generate the
   bounding boxes through other programs.
- The image has a top-left origin. X increases to the right, and Y increases
    downward.
- Return only the JSON object. Do **not** include any text, comments, or
   additional keys.
- If no objects of a category are detected, return an empty list for that
   category.

**Example output (structure only, placeholder values):**
{
"Platelets": [[...], [...]],
"RBC": [[...], [...]],
"WBC": [[...], [...]]
}
```

Listing 2. Detection prompt based on cell-name for benchmarking on BBBC dataset

```
################################################
# Detection Prompt for BBBC dataset #
################################################

You will be given a microscopic cell image from a stained blood smear. Your
   task is to detect and generate bounding boxes around the following three
   categories of blood components:

1. **gametocyte**: Typically crescent or banana-shaped.
2. **leukocyte**: Large, round or irregular shape, often with internal
   texture.
3. **schizont**: Round or oval, may appear as a cluster inside a red cell.
4. **red blood cell**: Smooth, round, and uniform with no internal
   structures.
5. **ring**: Small ring-like shape, usually inside a red cell.
6. **trophozoite**: Irregular shape with more solid or filled appearance
   than a ring.

Return **only** a JSON object with exactly these three keys: `gametocyte`, `
   leukocyte`, `schizont`, `red blood cell`, `ring`, `trophozoite`. Each key
    must map to a list of bounding boxes, with each bounding box defined as
   an array of four integers: `[x1, y1, x2, y2]` (pixel coordinates, where (
   x1, y1) is the top-left corner and (x2, y2) is the bottom-right corner).

**Important**:
- The image has a top-left origin. X increases to the right, and Y increases
    downward.
- Return only the JSON object. Do **not** include any text, comments, or
   additional keys.
- If no objects of a category are detected, return an empty list for that
   category.


You are forced to generate the bounding box and not allowed to return other
   suggestions or code to generate the bounding boxes through other programs.

**Example output (structure only, placeholder values):**
{
"gametocyte": [[...], [...]],
"leukocyte": [[...], [...]],
"schizont": [[...], [...]],
"red blood cell": [[...], [...]],
"ring": [[...], [...]],
"trophozoite": [[...], [...]]
```

```
}
```

Listing 3. Detection prompt based on cell-shape for benchmarking on NIH-3T3 dataset

```
###############################################
# Detection Prompt for NIH-3T3 dataset #
###############################################

You will be given a microscopic cell image from a NIH-3T3 microscopy. Your
   task is to get draw bounding boxes around the following three categories
   of cell shape components components:

1. **Round**: Cells appear circular, and sometimes with smooth edges.
2. **Spindle**: Cells are elongated and tapered, resembling a spindle or
   stretched ellipse.
3. **Polygonal**: Cells have multiple angles or sides.

Return **only** a JSON object with exactly these three keys: 'Round', '
   Spindle', 'Polygonal'. Each key must map to a list of bounding boxes,
   with each bounding box defined as an array of four integers: '[x1, y1, x2
   , y2]' (pixel coordinates, where (x1, y1) is the top-left corner and (x2,
    y2) is the bottom-right corner).

**Important**:
- Return the bounding box coordinate; you are not allowed to return other
   suggestions or code to generate the bounding boxes through other programs.

- The image has a top-left origin. X increases to the right, and Y increases
    downward.
- Return only the JSON object. Do **not** include any text, comments, or
   additional keys.
- If no objects of a category are detected, return an empty list for that
   category.

**Example output (structure only, placeholder values):**
{
"Round": [[...], [...]],
"Spindle": [[...], [...]],
"Polygonal": [[...], [...]]
}
```

Listing 4. Detection prompt based on cell-shape for benchmarking on Rat-C6 dataset

```
###############################################
# Detection Prompt for Rat-C6 dataset #
###############################################
```

```
You will be given a microscopic cell image. Your task is to detect and
   generate bounding boxes around the following three categories of cell
   shape components components:

1. **Round**: Cells appear circular, and sometimes with smooth edges.
2. **Spindle**: Cells are elongated and tapered, resembling a spindle or
   stretched ellipse.
3. **Polygonal**: Cells have multiple angles or sides.

Return **only** a JSON object with exactly these three keys: `Round`, `
   Spindle`, `Polygonal`. Each key must map to a list of bounding boxes,
   with each bounding box defined as an array of four integers: `[x1, y1, x2
   , y2]` (pixel coordinates, where (x1, y1) is the top-left corner and (x2,
    y2) is the bottom-right corner).

**Important**:
- Return the bounding box coordinate; you are not allowed to return other
   suggestions or code to generate the bounding boxes through other programs.

- The image has a top-left origin. X increases to the right, and Y increases
    downward.
- Return only the JSON object. Do **not** include any text, comments, or
   additional keys.
- If no objects of a category are detected, return an empty list for that
   category.

**Example output (structure only, placeholder values):**
{
"Round": [[12, 34, 56, 78], [91, 102, 110, 123]],
"Spindle": [[...], [...]],
"Polygonal": [[...]]
}
```

Listing 5. Classification prompt based on cell-name for benchmarking on BCCD dataset

```
################################################
# Classification Prompt for BCCD dataset #
################################################

You will be provided with an image crop of a microscopic cell. The cropped
   image dimensions are {} pixels, and it should contain only one cell.

**Your task is to classify the cell type into one of the following
   categories based on its visual characteristics:**
```

```
1. **Platelets**: These are small, irregularly shaped cell fragments with no
    nucleus. They typically appear much smaller than other cells.
2. **RBC** (Red Blood Cells): These are biconcave disc-shaped cells with
   smooth, round outlines. They generally lack a nucleus and have a uniform
   appearance.
3. **WBC** (White Blood Cells): These are larger cells with prominent, multi
   -lobed nuclei and more granular cytoplasm. They may have irregular shapes
    and internal complexity.
4. **None**: Any other shape, multiple cells, no visible cell, or ambiguous
   cases.

**Guidelines:**

- If the cell appears small and irregular with no nucleus, classify it as `
   Platelets`.
- If the cell has a smooth, round shape with no visible internal structure
   or nucleus, classify it as `RBC`.
- If the cell is larger, has a visible nucleus or complex internal structure
   , classify it as `WBC`.
- For any other cases, classify it as `None`.

**Response Format:**

- Return only the exact label (`Platelets`, `RBC`, `WBC`, or `None`) as a
   single string.
- Do not include any additional text, quotes, or formatting.
```

Listing 6. Classification prompt based on cell-name for benchmarking on BBBC dataset

```
###############################################
# Classification Prompt for BBBC dataset #
###############################################

You will be provided with an image crop of a microscopic cell from a stained
    blood smear. The cropped image dimensions are {} pixels, and it should
   contain only one cell or parasite.

**Your task is to classify the cell or parasite type into one of the
   following categories based on its shape and appearance:**

1. **gametocyte**: Typically crescent or banana-shaped.
2. **leukocyte**: Large, round or irregular shape, often with internal
   texture.
3. **schizont**: Round or oval, may appear as a cluster inside a red cell.
4. **red blood cell**: Smooth, round, and uniform with no internal
   structures.
```

```
5. **ring**: Small ring-like shape, usually inside a red cell.
6. **trophozoite**: Irregular shape with more solid or filled appearance
   than a ring.
7. **None**: Any other shape, multiple cells, no visible cell, or ambiguous
   cases.

**Guidelines:**

- If the cell matches the **gametocyte** description, classify it as `
   gametocyte`.
- If the cell matches the **leukocyte** description, classify it as `
   leukocyte`.
- If the cell matches the **schizont** description, classify it as `schizont
   `.
- If the cell matches the **red blood cell** description, classify it as `
   red blood cell`.
- If the cell matches the **ring** description, classify it as `ring`.
- If the cell matches the **trophozoite** description, classify it as `
   trophozoite`.
- If none of the above apply, or if the image is ambiguous, contains
   multiple cells, or no visible cell, classify it as `None`.

**Response Format:**

- Return only the exact label (`gametocyte`, `leukocyte`, `schizont`, `red
   blood cell`, `ring`, `trophozoite`, or `None`) as a single string.
- Do not include any additional text, quotes, or formatting.
```

Listing 7. Classification prompt based on cell-shape for benchmarking on NIH-3T3 dataset

```
################################################
# Classification Prompt for NIH-3T3 dataset #
################################################

You will be provided with an image crop of a microscopic cell from a NIH-3T3
    microscopy image. The cropped image dimensions are {} pixels, and it
   should contain only one cell.

**Your task is to classify the cell shape into one of the following
   categories based on its appearance:**

1. **Round**: Cells appear circular, and sometimes with smooth edges.
2. **Spindle**: Cells are elongated and tapered, resembling a spindle or
   stretched ellipse.
3. **Polygonal**: Cells have multiple angles or sides.
```

```
4. **None**: Any other shape, multiple cells, no visible cell, or ambiguous
   cases.

**Guidelines:**
- If the cell matches the **Round** description, classify it as `Round`.
- If the cell matches the **Spindle** description, classify it as `Spindle`.
- If the cell matches the **Polygonal** description, classify it as `
   Polygonal`.
- For any other cases, classify it as `None`.

**Response Format:**
- Return only the exact label (`Round`, `Spindle`, `Polygonal`, or `None`)
   as a single string.
- Do not include any additional text, quotes, or formatting.
```

Listing 8. Classification prompt based on cell-shape for benchmarking on Rat-C6 dataset

```
###############################################
# Classification Prompt for Rat-C6 dataset #
###############################################

You will be provided with an image crop of a microscopic cell from a
   microscopic image. The cropped image dimensions are {} pixels, and it
   should contain only one cell.

**Your task is to classify the cell shape into one of the following
   categories based on its appearance:**

1. **Round**: Cells appear circular, and sometimes with smooth edges.
2. **Spindle**: Cells are elongated and tapered, resembling a spindle or
   stretched ellipse.
3. **Polygonal**: Cells have multiple angles or sides.
4. **None**: Any other shape, multiple cells, no visible cell, or ambiguous
   cases.

**Guidelines:**
- If the cell matches the **Round** description, classify it as `Round`.
- If the cell matches the **Spindle** description, classify it as `Spindle`.
- If the cell matches the **Polygonal** description, classify it as `
   Polygonal`.
- For any other cases, classify it as `None`.

**Response Format:**
- Return only the exact label (`Round`, `Spindle`, `Polygonal`, or `None`)
   as a single string.
- Do not include any additional text, quotes, or formatting.
```

2 DATASET-WISE EVALUATION RESULTS

Table S1. Dataset-wise F1-score evaluation of YOLO11n under few-shot (K=6) fine-tuning, reported using average F1-score (mF1) across multiple benchmark datasets.

K = 6 Shot Setting					
**Method / Dataset**	**BCCD**	**BBBC/Malaria**	**NIH-3T3**	**RatC6/LIVECell**	**Avg mF1 ↑**
YOLO11n (Fine-tuned)	0.0610	0.0439	0.0071	0.0039	0.0289

Table S2. Evaluation results for few-shot object detection in the zero-shot setting. We report the mF1 score and Mean IoU (TP@0.5) for various VLMs on dataset BCCD.

**EXP1: Zero Shot-T**		
**Method**	**mF1 [.05:.70] ↑**	**Mean IoU (TP@0.5) ↑**
GroundingDINO	0.13	**0.79**
OWL-ViT	0.12	0.78
Gemini-2.5-Flash	0.11	0.58
Gemini-2.5-Flash-Thinking	**0.14**	0.61
Claude-3.7-Sonnet	0.05	0.00
Claude-3.7-Sonnet-Thinking	0.07	0.60

Table S3. Evaluation results for few-shot object detection across three conditions (K=1,3,6) for dataset BCCD

**Method**	**K=1**		**K=3**		**K=6**	
	**mF1 ↑**	**Mean IoU ↑**	**mF1 ↑**	**Mean IoU ↑**	**mF1 ↑**	**Mean IoU ↑**
**Few-Shot-V**						
OWL-ViT	**0.22 ± 0.0290**	**0.75 ± 0.0008**	**0.21 ± 0.0206**	**0.75 ± 0.0048**	**0.31 ± 0.0185**	**0.75 ± 0.0021**
**Few-Shot-MMD**						
GPT-4o	0.08 ± 0.0744	0.29 ± 0.4056	0.07 ± 0.0775	0.29 ± 0.4077	0.07 ± 0.0717	0.63 ± 0.0786
GPT-5	0.16 ± 0.0002	0.57 ± 0.0022	0.15 ± 0.0014	0.57 ± 0.1665	0.14 ± 0.0087	0.58 ± 0.0325
Gemini-2.5-Flash	0.08 ± 0.0247	0.59 ± 0.0082	0.07 ± 0.0498	0.60 ± 0.0149	0.09 ± 0.0195	0.61 ± 0.0134
Gemini-2.5-Flash-Thinking	**0.21 ± 0.2093**	**0.72 ± 0.1553**	**0.27 ± 0.3061**	**0.70 ± 0.1623**	**0.28 ± 0.3160**	**0.72 ± 0.1305**
Claude-3.7-Sonnet	0.05 ± 0.0141	0.17 ± 0.0495	0.05 ± 0.0177	0.17 ± 0.0778	0.05 ± 0.0226	0.59 ± 0.0120
Claude-3.7-Sonnet-Thinking	0.07 ± 0.0141	0.52 ± 0.0424	0.07 ± 0.0212	0.54 ± 0.1344	0.06 ± 0.0233	0.62 ± 0.0636
**Few-Shot-MMC**						
GPT-4o	0.48 ± 0.0019	0.83 ± 0.0027	0.51 ± 0.0243	0.83 ± 0.0067	0.53 ± 0.0066	**0.83 ± 0.0028**
GPT-5	**0.51 ± 0.0034**	0.82 ± 0.0006	**0.53 ± 0.0047**	0.82 ± 0.0013	**0.54 ± 0.0026**	0.82 ± 0.0011
Gemini-2.5-Flash	0.45 ± 0.0220	0.83 ± 0.0066	0.50 ± 0.0309	0.83 ± 0.0067	0.51 ± 0.0329	0.82 ± 0.0016
Gemini-2.5-Flash-Thinking	0.41 ± 0.0564	0.84 ± 0.0141	0.12 ± 0.0223	**0.85± 0.0200**	0.17 ± 0.0339	0.56 ± 0.0021
Claude-3.7-Sonnet	0.24 ± 0.0197	**0.84 ± 0.0002**	0.38 ± 0.0406	0.83 ± 0.0015	0.41 ± 0.0232	0.81 ± 0.0007
Claude-3.7-Sonnet-Thinking	0.23 ± 0.0314	0.82 ± 0.0067	0.43 ± 0.0323	0.82 ± 0.0124	0.39 ± 0.0373	0.81 ± 0.0020

Table S4. Evaluation results for few-shot object detection in the zero-shot setting. We report the mF1 score and Mean IoU (TP@0.5) for various VLMs on dataset BBBC.

**EXP1: Zero Shot-T**		
**Method**	**mF1 [.05:.70] ↑**	**Mean IoU (TP@0.5) ↑**
GroundingDINO	0.01	0.84
OWL-ViT	0.02	**0.87**
Gemini-2.5-Flash	0.02	0.60
Gemini-2.5-Flash-Thinking	**0.04**	0.60
Claude-3.7-Sonnet	0.02	0.00
Claude-3.7-Sonnet-Thinking	0.00	0.00

Table S5. Evaluation results for few-shot object detection across three conditions (K=1,3,6) for dataset BBBC

**Method**	**K=1**		**K=3**		**K=6**	
	**mF1 ↑**	**Mean IoU ↑**	**mF1 ↑**	**Mean IoU ↑**	**mF1 ↑**	**Mean IoU ↑**
**Few-Shot-V**						
OWL-ViT	**0.04 ± 0.0030**	**0.84 ± 0.0106**	**0.11 ± 0.0025**	**0.85 ± 0.0026**	**0.14 ± 0.0867**	**0.85 ± 0.0032**
**Few-Shot-MMD**						
GPT-4o	0.01 ± 0.0109	0.30 ± 0.4294	0.01 ± 0.0109	0.54 ± 0.0778	0.01 ± 0.0122	0.26 ± 0.3726
GPT-5	0.02 ± 0.0018	0.48 ± 0.0247	0.02 ± 0.0014	0.44 ± 0.1665	0.02 ± 0.0087	0.52 ± 0.0325
Gemini-2.5-Flash	0.01 ± 0.0092	0.55 ± 0.0066	0.01 ± 0.0039	0.32 ± 0.4586	0.01 ± 0.0062	**0.61 ± 0.0197**
Gemini-2.5-Flash-Thinking	**0.03 ± 0.0251**	**0.56 ± 0.0028**	**0.03 ± 0.0279**	**0.60 ± 0.0134**	**0.03 ± 0.0332**	0.59 ± 0.0172
Claude-3.7-Sonnet	0.02 ± 0.0141	0.17 ± 0.0495	0.02 ± 0.0177	0.17 ± 0.0778	0.02 ± 0.0226	0.44 ± 0.0120
Claude-3.7-Sonnet-Thinking	0.01 ± 0.0141	0.42 ± 0.0424	0.06 ± 0.0212	0.39 ± 0.1344	0.07 ± 0.0233	0.47 ± 0.0636
**Few-Shot-MMC**						
GPT-4o	0.19 ± 0.0125	0.86 ± 0.0020	0.22 ± 0.0198	0.86 ± 0.0022	0.25 ± 0.0189	0.86 ± 0.0019
GPT-5	**0.25 ± 0.0013**	0.86 ± 0.0006	**0.28 ± 0.0028**	0.85 ± 0.0010	**0.30 ± 0.0035**	0.85 ± 0.0007
Gemini-2.5-Flash	0.12 ± 0.0600	0.86 ± 0.0054	0.07 ± 0.0386	0.86 ± 0.0106	0.10 ± 0.0135	0.86 ± 0.0023
Gemini-2.5-Flash-Thinking	0.11 ± 0.0564	**0.87± 0.0141**	0.13 ± 0.0223	**0.86± 0.0200**	0.14 ± 0.0339	**0.86± 0.0021**
Claude-3.7-Sonnet	0.01 ± 0.0014	0.82 ± 0.0037	0.02 ± 0.0031	0.83 ± 0.0091	0.01 ± 0.0026	0.82 ± 0.0017
Claude-3.7-Sonnet-Thinking	0.01 ± 0.0086	0.42 ± 0.5899	0.01 ± 0.0112	0.42 ± 0.5885	0.01 ± 0.0104	0.42 ± 0.5897

Table S6. Evaluation results for few-shot object detection in the zero-shot setting. We report the mF1 score and Mean IoU (TP@0.5) for various VLMs on dataset NIH-3T3.

**EXP1: Zero Shot-T**		
**Method**	**mF1 [.05:.70] ↑**	**Mean IoU (TP@0.5) ↑**
GroundingDINO	0.01	0.51
OWL-ViT	0.00	0.00
Gemini-2.5-Flash	**0.06**	0.54
Gemini-2.5-Flash-Thinking	0.05	**0.62**
Claude-3.7-Sonnet	0.04	0.56
Claude-3.7-Sonnet-Thinking	0.05	0.54

Table S7. Evaluation results for few-shot object detection across three conditions (K=1,3,6) for dataset NIH-3T3

**Method**	**K=1**		**K=3**		**K=6**	
	**mF1 ↑**	**Mean IoU ↑**	**mF1 ↑**	**Mean IoU ↑**	**mF1 ↑**	**Mean IoU ↑**
**Few-Shot-V**						
OWL-ViT	**0.06 ± 0.0076**	**0.67 ± 0.0189**	**0.03 ± 0.0233**	**0.67 ± 0.0133**	**0.02 ± 0.0001**	**0.64 ± 0.0460**
**Few-Shot-MMD**						
GPT-4o	0.04 ± 0.0321	0.29 ± 0.4126	0.03 ± 0.0180	0.33 ± 0.4686	0.03 ± 0.0245	0.26 ± 0.3655
GPT-5	0.05 ± 0.0022	**0.64 ± 0.1007**	0.04 ± 0.0014	0.61 ± 0.1665	0.05 ± 0.0087	**0.70± 0.0325**
Gemini-2.5-Flash	0.04 ± 0.0046	0.58 ± 0.0913	0.05 ± 0.0192	0.52 ± 0.0283	0.05 ± 0.0028	0.29 ± 0.4053
Gemini-2.5-Flash-Thinking	**0.06 ± 0.0396**	0.33 ± 0.4706	**0.07 ± 0.0351**	**0.64 ± 0.0488**	0.06 ± 0.0444	0.64 ± 0.0682
Claude-3.7-Sonnet	0.04 ± 0.0141	0.54 ± 0.0495	0.04 ± 0.0177	0.17 ± 0.0778	0.05 ± 0.0226	0.61 ± 0.0120
Claude-3.7-Sonnet-Thinking	0.05 ± 0.0141	0.54 ± 0.0424	0.06 ± 0.0212	0.56 ± 0.1344	**0.06± 0.0233**	0.60 ± 0.0636
**Few-Shot-MMC**						
GPT-4o	**0.12 ± 0.0007**	0.69 ± 0.0122	**0.13 ± 0.0004**	**0.69 ± 0.0139**	**0.14 ± 0.0070**	0.69 ± 0.0066
GPT-5	0.11 ± 0.0051	**0.69 ± 0.0010**	0.11 ± 0.0037	0.69 ± 0.0029	0.12 ± 0.0059	0.69 ± 0.0003
Gemini-2.5-Flash	0.09 ± 0.0098	0.67 ± 0.0112	0.09 ± 0.0035	0.68 ± 0.0021	0.09 ± 0.0019	0.68 ± 0.0049
Gemini-2.5-Flash-Thinking	0.10 ± 0.0564	0.69 ± 0.0141	0.10 ± 0.0223	0.69 ± 0.0200	0.09 ± 0.0339	**0.69± 0.0021**
Claude-3.7-Sonnet	0.10 ± 0.0012	0.68 ± 0.0102	0.12 ± 0.0044	0.69 ± 0.0086	0.10 ± 0.0052	0.68 ± 0.0035
Claude-3.7-Sonnet-Thinking	0.10 ± 0.0015	0.68 ± 0.0085	0.11 ± 0.0044	0.69 ± 0.0035	0.09 ± 0.0058	0.68 ± 0.0015

Table S8. Evaluation results for few-shot object detection in the zero-shot setting. We report the mF1 score and Mean IoU (TP@0.5) for various VLMs on dataset Rat-C6.

**EXP1: Zero Shot-T**		
**Method**	**mF1 [.05:.70] ↑**	**Mean IoU (TP@0.5) ↑**
GroundingDINO	0.00	0.00
OWL-ViT	0.01	0.00
Gemini-2.5-Flash	0.01	0.57
Gemini-2.5-Flash-Thinking	0.01	0.57
Claude-3.7-Sonnet	**0.02**	**0.79**
Claude-3.7-Sonnet-Thinking	0.01	0.00

Table S9. Evaluation results for few-shot object detection across three conditions (K=1,3,6) for dataset RatC6

**Method**	**K=1**		**K=3**		**K=6**	
	**mF1 ↑**	**Mean IoU ↑**	**mF1 ↑**	**Mean IoU ↑**	**mF1 ↑**	**Mean IoU ↑**
**Few-Shot-V**						
OWL-ViT	**0.01** ± **0.0096**	**0.32** ± **0.4471**	**0.01** ± **0.0014**	**0.59** ± **0.0615**	**0.01** ± **0.0038**	**0.61** ± **0.0501**
**Few-Shot-MMD**						
GPT-4o	0.01 ± 0.0176	0.28 ± 0.4016	0.01 ± 0.0130	0.31 ± 0.4439	0.01 ± 0.0127	0.26 ± 0.3744
GPT-5	0.03 ± 0.0008	0.58 ± 0.0416	0.02 ± 0.0014	0.44 ± 0.1665	0.03 ± 0.0087	**0.57**± **0.0325**
Gemini-2.5-Flash	0.01 ± 0.0033	0.53 ± 0.0778	0.01 ± 0.0083	0.25 ± 0.3575	0.01 ± 0.0013	0.55 ± 0.0990
Gemini-2.5-Flash-Thinking	**0.08** ± **0.0993**	0.39 ± 0.5571	**0.08** ± **0.1010**	0.39 ± 0.5536	**0.08** ± **0.0858**	0.39 ± 0.5546
Claude-3.7-Sonnet	0.02 ± 0.0141	0.17 ± 0.0495	0.01 ± 0.0177	**0.58**± **0.0778**	0.02 ± 0.0226	0.57 ± 0.0120
Claude-3.7-Sonnet-Thinking	0.02 ± 0.0141	**0.59** ± **0.0424**	0.01 ± 0.0212	0.39 ± 0.1344	0.02 ± 0.0233	0.52 ± 0.0636
**Few-Shot-MMC**						
GPT-4o	**0.19** ± **0.0124**	0.78 ± 0.0064	**0.19** ± **0.0199**	**0.79** ± **0.0060**	**0.18** ± **0.0089**	0.79 ± 0.0078
GPT-5	0.14 ± 0.0010	0.79 ± 0.0018	0.14 ± 0.0046	0.79 ± 0.0008	0.14 ± 0.0043	0.79 ± 0.0044
Gemini-2.5-Flash	0.13 ± 0.0216	**0.79** ± **0.0031**	0.12 ± 0.0312	0.79 ± 0.0060	0.11 ± 0.0229	0.79 ± 0.0009
Gemini-2.5-Flash-Thinking	0.14 ± 0.0564	0.78 ± 0.0141	0.14 ± 0.0223	0.79 ± 0.0200	0.11 ± 0.0339	0.79 ± 0.0021
Claude-3.7-Sonnet	0.11 ± 0.0026	0.78 ± 0.0141	0.10 ± 0.0038	0.79 ± 0.0031	0.07 ± 0.0031	0.78 ± 0.0062
Claude-3.7-Sonnet-Thinking	0.09 ± 0.0008	0.78 ± 0.0019	0.10 ± 0.0022	0.79 ± 0.0159	0.08 ± 0.0401	**0.80**± **0.0144**

3 SIGNIFICANCE TEST RESULTS

Table S10. Paired t-test comparing K=1 vs K=6 mF1 across models. For each model, we report the mean difference $\Delta = \bar{X}_{K=6} - \bar{X}_{K=1}$ (positive = improvement with more shots), which quantifies the magnitude of the effect; the t-statistic, which measures the size of Δ relative to its trial-level variability (larger $|t|$ indicates a more reliable effect); and the one-sided p-value testing H_1: $\mu_{K=6} > \mu_{K=1}$, i.e., whether additional few-shot support significantly improves detection. A small p-value indicates that the observed improvement is unlikely to be due to sampling noise alone. With $n = 3$ trials per condition, Δ and t should be read alongside p. Significance levels: $^{*}p < 0.1$, $^{**}p < 0.05$, $^{***}p < 0.01$.

Setting	Model	Δ mF1	t	p-value (1-sided)
Few-Shot-V	OWL-ViT	+0.090	+12.28	0.0033***
Few-Shot-MMD	GPT-4o	+0.020	+1.12	0.189
	GPT-5	+0.020	+3.60	0.0347**
	Gemini-2.5-Flash	+0.020	+1.09	0.195
	Gemini-2.5-Flash-Thinking	+0.040	+1.71	0.115
	Claude-3.7-Sonnet	+0.010	+1.55	0.131
	Claude-3.7-Sonnet-Thinking	+0.020	+3.42	0.0379**
Few-Shot-MMC	GPT-4o	+0.050	+7.30	0.0091***
	GPT-5	+0.020	+48.04	0.0002***
	Gemini-2.5-Flash	+0.010	+2.36	0.071*
	Gemini-2.5-Flash-Thinking	+0.010	+0.36	0.376
	Claude-3.7-Sonnet	+0.030	+0.98	0.216
	Claude-3.7-Sonnet-Thinking	+0.070	+2.39	0.070*

Table S11. Paired t-test comparing "thinking" vs "non-thinking" variants on mF1, pooled across K=1 and K=6 trials. For each model pair, we report the mean difference $\Delta = \bar{X}_{\text{thinking}} - \bar{X}_{\text{non-thinking}}$ (positive = thinking variant performs better), which quantifies the magnitude of the effect; the t-value, which measures the size of Δ relative to its trial-level variability (larger $|t|$ indicates a more reliable effect); and the one-sided p-value testing H_1: $\mu_{\text{thinking}} > \mu_{\text{non-thinking}}$, i.e., whether the reasoning variant significantly outperforms its non-reasoning counterpart. A small p-value indicates that the observed difference is unlikely to be due to sampling noise alone. Significance levels: $^{*}p < 0.1$, $^{**}p < 0.05$, $^{***}p < 0.01$.

Setting	Model pair	Δ mF1	t	p-value (1-sided)
Few-Shot-MMD	Gemini-2.5-Flash	+0.150	+8.52	0.0002***
	Claude-3.7-Sonnet	+0.015	+1.15	0.151
Few-Shot-MMC	Gemini-2.5-Flash	−0.050	−2.78	0.981
	Claude-3.7-Sonnet	+0.010	+0.40	0.354
